# Genome Sequence of the Polycyclic Aromatic Hydrocarbon-Degrading Bacterium Strain *Marinobacter nanhaiticus* D15-8W^T^

**DOI:** 10.1128/genomeA.00301-13

**Published:** 2013-05-30

**Authors:** Zhisong Cui, Wei Gao, Qian Li, Guangsu Xu, Li Zheng

**Affiliations:** Marine Ecology Research Center, the First Institute of Oceanography, State Oceanic Administration of China, Qingdao, China

## Abstract

*Marinobacter nanhaiticus* strain D15-8W^T^ was isolated from a phenanthrene-degrading consortium, enriched from sediment of the South China Sea. Here, we present the draft genome of strain D15-8W^T^, which contains 5,358,309 bp with a G+C content of 58.53% and contains 4,829 protein-coding genes and 47 tRNA genes.

## GENOME ANNOUNCEMENT

Some bacteria of the genus *Marinobacter* are likely to be actively involved in the decomposition processes of marine contaminants, including polycyclic aromatic hydrocarbon (PAHs). At least five species of this genus with validly published names were reported with regard to their ability to degrade aliphatic and aromatic hydrocarbons (1–6). *Marinobacter nanhaiticus* strain D15-8W^T^ was isolated from a sediment sample from the South China Sea (lat 19.98, long 111.42; depth 69 m) for its ability to use PAHs as the sole carbon and energy source. *M. nanhaiticus* D15-8W^T^ is a Gram-negative, rod-shaped, slightly halophilic, and facultatively anaerobic bacterium. It uses naphthalene, phenanthrene, and anthracene as sole carbon sources. The complete genome sequences of two aliphatic hydrocarbon-degrading *Marinobacter* species, *M. algicola* DG893 (GenBank accession number ABCP00000000) and *M. aquaeolei* VT8 (GenBank accession numbers CP000514 to CP000516), have already been published; nevertheless, a genome sequence for the aromatic hydrocarbon-degrading species *M. nanhaiticus* D15-8W^T^ might aid in advancing our understanding of the capacity of *Marinobacter* species for organic pollutant degradation in marine environments.

The genome sequence of *M. nanhaiticus* D15-8W^T^ was determined by BGI (Shenzhen, China) using Solexa paired-end sequencing technology. A total of 9,777,780 paired-end reads (500-bp and 2,000-bp libraries) were generated to reach a 164-fold depth of coverage with an Illumina HiSeq 2000 (Illumina Inc., San Diego, CA). The reads were assembled using SOAPdenovo (Ver. 1.05) (7, 8). The resulting genome sequence of *M. nanhaiticus* D15-8W^T^ consists of 14 contigs (N_90_, 530,102) of 5,358,309 bp and has an average G+C content of 58.53%. Gene annotation was carried out by the NCBI Prokaryotic Genomes Automatic Annotation Pipeline (PGAAP) (http://www.ncbi.nlm.nih.gov/genomes/static/Pipeline.html), which was followed by manual editing. The genome contains 4,765 candidate protein-encoding genes (with an average size of 980 bp), giving a coding intensity of 87.15%. A total of 3,872 proteins were assigned to cluster of orthologous groups (COG) families. A total of 47 tRNA genes for all 20 amino acids and 1 16S-23S-5S rRNA operon were identified by tRNAscan (Ver. 1.23) (9) and rRNAmmer (Ver. 1.2) (10).

In particular, we analyzed the genes possibly responsible for PAH degradation. Each gene encoding the alpha subunit and the ferredoxin subunit of ring-hydroxylating dioxygenase was found in the genome of *M. nanhaiticus* D15-8W^T^. Moreover, one gene encoding the extradiol ring-cleavage dioxygenase was also found in the genome sequence. The D15-8W^T^ genome sequence and its curated annotation are important assets to improve our understanding of the physiology and metabolic potential of *M. nanhaiticus* and will open up new opportunities related to the functional genomics of this species.

### Nucleotide sequence accession numbers.

The nucleotide sequence comprising the *Marinobacter nanhaiticus* strain D15-8W^T^ genome was deposited at DDBJ/EMBL/GenBank under the accession number APLQ00000000 (chromosome). The version described in this paper is the first version, APLQ01000000.
